# Editorial: The Role of Cellular Senescence in Health and Disease

**DOI:** 10.3389/fncel.2022.882417

**Published:** 2022-03-30

**Authors:** Ignacio Palmero, Vassilis Gorgoulis, Isabel Varela-Nieto

**Affiliations:** ^1^Cell Senescence and Tumor Suppression Lab, Institute of Biomedical Research “Alberto Sols” CSIC-UAM, Madrid, Spain; ^2^Molecular Carcinogenesis Group, Department of Histology and Embryology, Faculty of Medicine, National and Kapodistrian University of Athens, Athens, Greece; ^3^Biomedical Research Foundation, Academy of Athens, Athens, Greece; ^4^Division of Cancer Sciences, Faculty of Biology, Medicine and Health, School of Medical Sciences, University of Manchester, Manchester, United Kingdom; ^5^Center for New Biotechnologies and Precision Medicine, National and Kapodistrian University of Athens, Athens, Greece; ^6^Faculty of Health and Medical Sciences, University of Surrey, Guildford, United Kingdom; ^7^Neurobiology of Hearing Lab, Institute of Biomedical Research “Alberto Sols” CSIC-UAM, Madrid, Spain; ^8^Rare Diseases Networking Biomedical Research Centre (CIBERER), CIBER, Carlos III Institute of Health, Madrid, Spain; ^9^Hospital La Paz Institute for Health Research (IdiPAZ), Madrid, Spain

**Keywords:** cellular senescence, pathology, development, senotherapy, fibrosis, neurodegeneration, aging

Cellular senescence is a stable anti-proliferative state, which has an essential role in cell balance control in diverse physiological and pathological settings (Chan and Narita, 2019; Gorgoulis et al., 2019). Senescence research is a highly dynamic field that has experienced a radical expansion over the last few years with the identification of the role of senescence in a growing list of diseases and physiological processes and the promise for therapeutic interventions based on senescence (Munoz-Espin and Serrano, 2014; Paez-Ribes et al., 2019). The current Research Topic aims to give an overview of the latest advances in this field highlighting the progress in understanding the mechanism of senescence and its link to disease in the nervous system and other organs. The issue includes a wide range of articles, including original research reports, mini-reviews, and reviews that explore diverse angles of this topic, showcasing the current trends in senescence research.

Several contributions in this issue explore the role of senescence in the various components of the nervous system and its involvement in neurodegeneration. Martínez-Cué and Rueda present a comprehensive review that delves into the topic of the contribution of cellular senescence to the pathophysiology of neurodegenerative diseases. The review discusses both molecular mechanisms involved and the evidence that associates cellular senescence with several neurodegenerative disorders including Alzheimer's Disease (AD), Down syndrome, or Parkinson's Disease. Contributing to the discussion of senescence in neurodegeneration, Walton et al. review and discuss the evidence indicating that Aβ oligomers and the process of cellular senescence may be inextricably linked, at least in the early stages of AD. The role of Aβ oligomers as a senescence inducer and the possible role of secondary senescence in AD progression suggest that cellular senescence could possibly be an essential component of the pathological cascade of events within the amyloid cascade hypothesis rather than a separate etiology for AD progression. In their mini-review, Papadopoulos et al. explore another side of neurodegeneration and its relation to cellular senescence, this time with a focus on progressive multiple sclerosis. They review data suggesting involvement of cellular senescence in a wide variety of processes regulating disease progression, such as its role in chronic, non-remitting inflammation, in altered neuronal and glial cell function, in failure of remyelination and in impaired blood-brain barrier integrity. Finally, the possible neuroprotective role of senolytic and senomorphic treatments is discussed. As a mechanistic and methodological complement to these reviews, this special issue also includes an article in the form of a mini-review by Levstek et al., which discusses the topic of telomere attrition in neurodegenerative disorders, highlighting the promises and challenges of telomere studies in this context.

Also within the subject of neurobiology, two articles focus on the role of senescence in the auditory system. The article by Magariños et al. deepens our understanding of developmental senescence, highlighting the roles of TGFβ2, and cellular senescence in the regulation of cell fate within the developing inner ear and how this link is highly cell-type dependent. Interestingly, the study shows that TGFβ2 exerts a powerful action in inner ear neurogenesis but, contrary to other embryonic otic cell types, these effects are independent of cellular senescence. The study of cellular senescence in auditory cells is also addressed, this time in the context of aging of mechanotransducer cells, by Lin et al. These authors address the interesting relationship between mitohormesis and cellular senescence by identifying DRP-1 as a possible connector node between the two. They suggest a novel mechanism where DRP-1-induced mitophagy may reduce mitochondrial dysfunction and oxidative stress, thus blunting cellular senescence during aging of otic cells.

Among the pathologies linked to senescence, fibrotic diseases in different organs have attracted a lot of interest. In this issue, Lin and Xu discuss the roles of aging and cellular senescence in idiopathic pulmonary fibrosis, one of the diseases where the pathogenic role of senescence has been more extensively studied. In their review article, the authors focus on the molecular mechanisms by which senescent lung fibroblasts influence this fibrotic disease and the potential for senescence-based interventions in this pathology. Also in this context, Wu et al. review the role of cell cycle deregulation, a hallmark of the senescent phenotype, in renal fibrosis. As in other organs, the link between senescence and fibrosis in the kidney is complex. As highlighted by the authors, injury-associated senescence in tubular epithelial cells may promote fibrosis in a paracrine mechanism mediated by the SASP (Senescence-Associated Secretory Phenotype). However, the specific functional impact of cell cycle deregulation and senescence in renal fibrosis is likely to be more intricate, being influenced by factors like the profibrotic stimulus, renal cell types involved and the distinction between acute and chronic senescence.

Two original reports in this issue provide insights about additional lung diseases, besides fibrosis. In their *in silico* study, Dong et al. identify the role of senescence-associated genes TNFRSF12A and CD38 in chronic obstructive pulmonary disease (COPD), and discuss their contribution to chronic inflammation and accelerated aging that characterize COPD. Through functional pathway enrichment analysis they propose a model where stimulation of TWEAK/TNFRSF12A signaling enhances lung tissue remodeling, while induction of CD38 protein expression contributes to the accelerated aging observed in COPD patients. It is speculated that the balance between TNFRSF12A and CD38 proteins could play a major role in establishing a cycle of unresolvable tissue remodeling in COPD patients' lungs. Pan et al. explore the role of microRNA-221 in the pathogenesis of asthma. They show that inhibition of miR-221 in a murine asthma model reduces airway mucus metaplasia, inflammation, and remodeling. At the same time, miR-221 was found to regulate collagen deposition in the extracellular matrix through the PI3K-AKT pathway, shedding interesting light on miR-221 functions, and highlighting this microRNA as a potential therapeutic target.

A complementary perspective on cellular senescence and age-associated conditions is given in the review article by Li et al. The effects of SIRT6 as an inhibitor of cellular senescence and cell aging, and as an important player in cardiovascular disease through regulation of triglyceride synthesis and cholesterol homeostasis are extensively discussed. Possible clinical applications to target activation of SIRT6 as potential means to delay aging and treat cardiovascular diseases are explored.

Regarding the mechanism of senescence, the article by Shimoni et al. proposes a novel stimulus to add to the list of alterations in cellular homeostasis that have been shown to trigger the senescence response. These include genotoxic damage, oncogene activation, or mitochondrial dysfunction, to name a few. In this article, the authors report that bovine umbilical cord mesenchymal stem cells exposed to mild heat stress acquire features of the senescent phenotype, including cell-cycle arrest and lysosomal Senescence-Associated Beta Galactosidase activity. This phenotype seems to be associated with oxidative stress and mitochondrial damage. It would be interesting to characterize in more detail this response to understand the potential link between abnormal temperature conditions and senescence. Also, the article by Zhou et al. explores the link between cell senescence, intestinal epithelial integrity and gut microbiota. Using a mouse model of loss of function of Bmi-1, an epigenetic repressor of senescence, the authors show that Bmi-1 preserves intestinal epithelial barrier function and microbiota balance by preventing p16Ink4a-mediated senescence, in a process that apparently involves a non-canonical role of the p16Ink4a protein in tight junctions.

One of the major breakthroughs in senescence research in recent years has been the discovery of therapeutical interventions based in senescence. In their review in this issue, Thoppil and Riabowol provide an overview of the existing toolkit of compounds with the ability to selectively kill senescent cells (senolytics) or modify the senescent secretome (senomorphs), as well as senescent cell-specific drug delivery strategies, highlighting their therapeutic potential. The review by Kaur et al. explores a different angle of this subject, discussing alternative strategies to prevent age-induced tissue senescence based in the use of bioactive compounds from plants or food, known as nutraceuticals. Excitingly, some of these synthetic or natural senotherapeutic compounds have already entered clinical trials, opening the prospect to exploit senescence in the clinic.

In summary, we believe that the articles in this issue provide a timely and comprehensive overview of the current research efforts in the senescence field. As editors, we are grateful to the authors for their contributions and we hope that this Research Topic succeeds to convey to the readers the interest and promise of the studies on cellular senescence and its connection with human disease.

## Author Contributions

All authors contributed to writing and editing the manuscript.

## Funding

Research in the authors' laboratories was supported by the following grants: RTI2018-098520-B-I00 from the Spanish MCIN/AEI/10.13039/501100011033 and FEDER to IP. 2020ΣE01300001 from the Ministry of Development and Investment, 775 (Hippo) and 3782 (PACOREL) from the Hellenic Foundation for Research and Innovation, 70/3/8916 from NKUA-SARG, the Welfare Foundation for Social & Cultural Sciences (KIKPE), and H. Pappas donation to VG, PID2020-115274RB-I00 from the Spanish MCIN/AEI/10.13039/501100011033 and FEDER to IV-N. IP and IV-N were members of the Spanish Senescence Network, Senestherapy (RED2018-102698-T).

## Conflict of Interest

The authors declare that the research was conducted in the absence of any commercial or financial relationships that could be construed as a potential conflict of interest.

## Publisher's Note

All claims expressed in this article are solely those of the authors and do not necessarily represent those of their affiliated organizations, or those of the publisher, the editors and the reviewers. Any product that may be evaluated in this article, or claim that may be made by its manufacturer, is not guaranteed or endorsed by the publisher.

## References

[B1] Chan A. S. L. Narita M. (2019). Short-term gain, long-term pain: the senescence life cycle and cancer. Genes Dev. 33, 127–143. 10.1101/gad.320937.118 30709901 PMC6362810

[B2] Gorgoulis V. Adams P. D. Alimonti A. Bennett D. C. Bischof O. Bishop C.. (2019). Cellular senescence: defining a path forward. Cell 179, 813–827. 10.1016/j.cell.2019.10.005 31675495

[B3] Munoz-Espin D. Serrano M. (2014). Cellular senescence: from physiology to pathology. Nat. Rev. Mol. Cell Biol. 15, 482–496. 10.1038/nrm3823 24954210

[B4] Paez-Ribes M. Gonzalez-Gualda E. Doherty G. J. Munoz-Espin D. (2019). Targeting senescent cells in translational medicine. EMBO Mol. Med. 11:e10234. 10.15252/emmm.201810234 31746100 PMC6895604

